# Personalization of Electric Vehicle Accelerating Behavior Based on Motor Torque Adjustment to Improve Individual Driving Satisfaction

**DOI:** 10.3390/s21123951

**Published:** 2021-06-08

**Authors:** Haksu Kim

**Affiliations:** Future Technology Division, ControlWorks, Seoul 06222, Korea; haksu.kim@control-works.co.kr

**Keywords:** personalized EV performance, individual driver behavior modeling, driving satisfactory, EV control, traction motor control

## Abstract

As worldwide vehicle CO_2_ emission regulations have been becoming more stringent, electric vehicles are regarded as one of the main development trends for the future automotive industry. Compared to conventional internal combustion engines, electric vehicles can generate a wider variety of longitudinal behaviors based on their high-performance motors and regenerative braking systems. The longitudinal behavior of a vehicle affects the driver’s driving satisfaction. Notably, each driver has their own driving style and as such demands a different performance for the vehicle. Therefore, personalization studies have been conducted in attempts to reduce the individual driving heterogeneity and thus improve driving satisfaction. In this respect, this paper first investigates a quantitative characterization of individual driving styles and then proposes a personalization algorithm of accelerating behavior of electric vehicles. The quantitative characterization determines the statistical expected value of the personal accelerating features. The accelerating features include physical values that can express acceleration behaviors and display different tendencies depending on the driving style. The quantified features are applied to calculate the correction factors for the target torque of the traction motor controller of electric vehicles. This driver-specific correction provides satisfactory propulsion performance for each driver. The proposed algorithm was validated through simulations. The results show that the proposed motor torque adjustment can reproduce different acceleration behaviors for an identical accelerator pedal input.

## 1. Introduction

### 1.1. Research Background and Motivation

**Figure 1:**
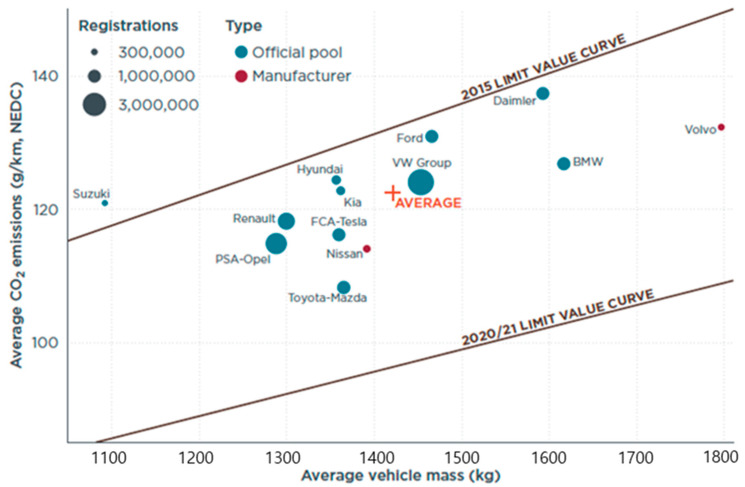
Performance of top-selling EU passenger car manufacturers in 2019 compared to 2015, and the 2020/2021 emissions target compliance curves.

In recent years, a central concern for the automotive industry has been the fact that CO_2_ emission regulations are becoming more stringent worldwide [1,2]. Figure 1 plots several car manufacturers’ average CO_2_ emissions in 2019 compared to 2015, as well as the 2020/2021 target values [3]. While the target emissions have decreased by 27% over the five-year period, the emission performance of actual passenger cars in 2019 is far below the upcoming target regulations. Moreover, in 2019, the European Council agreed on CO_2_ emission standards for new vehicles produced after 2020 [4]. In 2030, the average CO_2_ emissions of new passenger cars should be 37.5% lower than in 2021.

**Figure 2:**
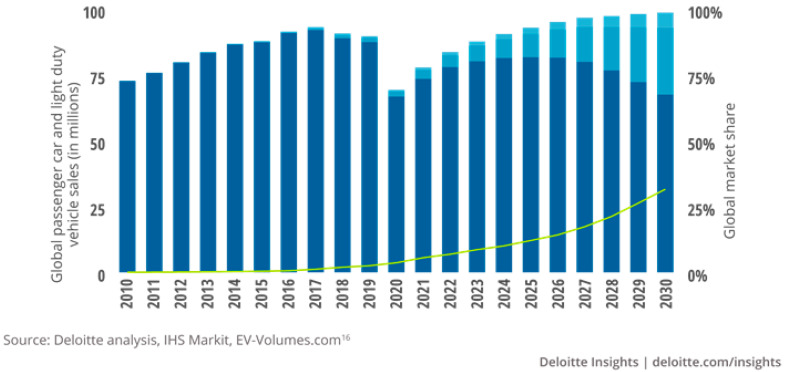
Outlook for annual global passenger-car and light-duty vehicle sales, up to 2030.

The ever-intensifying regulations pertaining to emissions and fuel economy have resulted in the recent growth of the Electric Vehicle (EV) market [5,6,7,8,9]. To reduce emissions, automakers have spurred the development of eco-friendly vehicles that provide cleaner, more efficient, and safer performance, such as Battery-powered Electric Vehicles (BEVs), Hybrid Electric Vehicles (HEVs), and Fuel-Cell Vehicles (FCVs) [10,11,12]. As shown in Figure 2, the EV share has increased in the global passenger car and light-duty vehicle market over the past ten years [13]. The EV market share is eventually expected to account for more than 25% of the market by 2030. Currently, BEVs already surpass HEVs’ global sales, and are predicted to account for 81% of all new EVs sold by 2030. In addition, due to the EV development having a lower barrier to entry compared to internal combustion engine technologies, the number of EV startups has increased. Currently, the success of Tesla, which now accounts for the majority of EV sales, is accelerating the growth of EV startups [14].

The relatively large number of early adopters of EVs makes it a suitable topic for personalization research. There are various factors that affect consumers’ adoption of EVs [15,16,17,18,19,20]. In studies on consumer EV adoption, factors affecting the adoption of EVs included consumer perceptions of the environment, fuel efficiency, and the fact that new types of vehicles—especially EVs—can influence decisions to buy EVs. Such research provided some understanding of individuals’ perceptions of EVs and the personal requirements needed to adopt a new type of car. Notably, other potential customers have resisted purchasing EVs due to the difference in longitudinal driving feel caused by the differences in the powertrain structure of EVs and Internal Combustion Engine Vehicles (ICEVs). As a result, the study of EVs needs to include an investigation of the driver’s personal satisfaction, not just research on how to enhance performance.

### 1.2. Problem Statements and Related Works

#### 1.2.1. Personalization in the Automotive Field

Personalization in the automobile field is still a relatively recent research issue. Customization of the automobile exterior, as well as personalization of the position of the driver’s seat and side mirrors are common issues. Currently, EVs have two main application areas of personalization: the personalization of driving performance and the personalization of driver assistance systems [21,22,23,24,25,26,27,28].

Personalization of products, which means changing something or designing a component for someone specific, is not a new concept, but advances in information and web technologies have generated considerable interest in various fields over the past 20 years. It allows service and product providers to extend personalization from one-on-one personal relationships with individual customers or users to automated services based on the analysis of data collected according to the personal tastes and behaviors of numerous users [29].

The general concept of personalization is intuitive, but the understanding and goals of personalization vary among different disciplines and researchers. Here, we will focus on personalization in terms of human–machine interaction and see this interaction as a means of making skills more acceptable and useful for people. This is particularly important in the field of advanced driver assistance systems, where the goal is not only to improve the driving experience, but also to improve driving safety and prevent accidents caused by human error.

Since such a system can only be effective when used in practice, it becomes necessary to gain the driver’s confidence, meet expectations for driving behavior, and not annoy the driver with irrelevant recommendations and precautions. However, driver expectations vary from driver to driver and within individual drivers, depending on the driver’s condition and the driving situation. Therefore, driver assistance and related systems appear to be particularly suitable for personalization.

In general, personalization is classified as either explicit or implicit personalization. Explicit personalization requires users to explicitly change their system by specifying their preferences and choosing the specific system settings that work best for them. Such an adaptive system limits the user’s choices to the system’s suggestions, but allows direct control. The main drawback of these systems includes limitations in the number of settings that the user can intuitively understand. For more complex support functions, it is very difficult to design settings, for example, as potential stings can go far beyond simple sporty and comfortable driving modes. In addition, the combined effect of setting several parameters at the same time is very opaque to the user. Conversely, implicit personalization observes a user’s behavior without directly requesting preferences from the user and derives a user model for predicting user preferences or behaviors based on these user data.

Over the years, most of the implicit personalization research in the automotive field has been conducted to improve the Advanced Driver Assistance System (ADAS). ADAS was conventionally designed to guarantee a level of general performance that satisfies unspecified individuals. However, an assistance system that is optionally used by a specific user, such as Adaptive Cruise Control (ACC), must satisfy a user’s personal preferences [30,31,32,33,34]. As a result, the personalization of ADAS has been researched to consider the driver’s specific personalities and to ensure a better use experience.

Most previous studies pertaining to practical applications of personalization have proposed a set of predefined values by categorizing drivers into several groups [35,36,37,38]. For example, three predefined clusters for acceleration profile parameters were proposed. According to the driver, one of the three clusters is selected in order to change the target acceleration profile characteristics. Furthering this selection, Rosenfeld et al. [36] classified drivers into the three groups through a large data set and using a regression model and a decision tree. However, it is difficult to reflect the group-based predefined sets of driving styles for each driver due to the great diversity of individual preferences and the fact that drivers might change their driving styles depending on the driving conditions.

Therefore, several studies have proposed driver models that directly map vehicle states through deterministic equations into control inputs, such as speed, acceleration, and pedal operation, based on an individual’s driving style [26,39,40,41]. In recent years, with the growth of computing devices and the rise of big data research, many researchers have applied data-driven learning models to driver behavior predictions. Markov models stochastically predict driving states for the uncertainties of real-driving situations [42,43,44]. Deep neural network models [45,46,47,48] have also been popular because they can provide accurate prediction performance and handle a large amount of data. However, neither of these methods are suitable for use in real-time embedded systems.

This paper primarily focuses on approaches for personalizing EV driving performance, which belongs to the category of implicit personalization, in order to model users through driver behavior observations.

#### 1.2.2. Personalization of EV Driving Performance

Compared to conventional ICEVs, it is relatively easy to personalize the performance of EVs. In particular, there are distinct differences in terms of longitudinal behavior. The longitudinal movement can be roughly divided into acceleration and deceleration. In the case of acceleration, it is easier to secure a higher performance in EVs than ICEVs. EVs consist of a much simpler powertrain and have an inherent advantage, as they use an electric motor instead of a standard engine. The electric drive motor has a wide range of performance coverages, with a fast response and high torque at low speed. In addition, as the transmission becomes unnecessary, linear control is possible, so performance adjustments are easier to apply. In the case of deceleration, a regenerative braking system can be used in addition to hydraulic brakes in EVs. The regenerative braking system is capable of adjusting its strength, enabling various deceleration performances to be realized.

In general, driving mode functions are offered in mass-production EVs. These functions typically have three or more options, and the EV’s deceleration performance is adjusted according to the option selected by the driver. The options are determined within the range of vehicle performance and are individually tuned by the developer during development. However, because the number of options is limited, they cannot reflect all the preferences for all drivers.

To add variety to these options, a new technology was proposed to allow drivers to customize the vehicle’s performance themselves [49,50]. The adjustable factors include seven performance features: the maximum torque output of the motor, ignition, acceleration and deceleration abilities, regenerative braking capacity, maximum speed limit, responsiveness, and energy use on climate control. The reasons for adjusting the degrees of acceleration and deceleration are diverse, and the fact that the levels can be subdivided further increases the degree of freedom in reflecting drivers’ characteristics. However, this system also belongs to an explicit form of personalization and requires the direct selection of the driver. In addition, the selection process for the all factors can become a more difficult problem for drivers to resolve, especially as the options become more and more complex.

### 1.3. Research Objectives and Scope

This paper proposes the personalization of the accelerating performance of EVs in a driver’s manual driving condition. The fundamental process for personalization is to model the driving behaviors based on driver-specific data [51,52,53,54]. The individual driving data is then applied to change the driving style difference among multiple drivers. Therefore, real-driving data were analyzed to quantify individual driving behaviors. Since the target application of this research is to tune the EV’s acceleration performance according to only driver characteristics, data analysis was conducted in free-driving scenarios that the driver can drive at his own will without any interruption.

The EV powertrain control system is personalized based on driver characteristics. General EV powertrain control systems utilize look-up tables for the target torque, which have been calibrated through propulsion motor experiments. The values of the look-up tables are commonly designed to guarantee the stable acceleration of EVs. Notably, these values can change the acceleration performance of the vehicle through corrections. As such, if the correction is performed according to an individual driver’s needs, driving satisfaction for each driver can be increased [55,56,57]. The proposed personalization algorithm for the EV powertrain control system determines the correction factors according to the driver’s characteristics. These characteristics are quantified through the real driving data of individual drivers and then correlated to the correction factors. As a result, the compensated target torque provides a different acceleration performance for each driver. The proposed personalized powertrain control system was subsequently validated through simulations using IPG CarMaker, which provides a reliable environment for automotive research. The simulation results were then compared with actual driving data to show their similarities with actual driver behavior.

To clarify the contribution of this study, the featured aspects of this study are summarized as follows. We propose a correction method for the target torque calculated using the look-up table of the motor rotation speed and the accelerator pedal position under the condition of manual driving. The target torque table was calibrated through repetitive experiments to provide standard acceleration performance; previous studies added correction look-up tables based on vehicle specifications to compensate for the target torque. The limitations of these approaches were that the correction look-up tables consider only the target motor’s own operation range and provide discrete correction results. To overcome these limitations, the proposed method determines correction factors based on personal driving features, such as the target driver’s preferred maximum acceleration and maximum jerk. To ensure stability, the proposed correction factor can reflect various driver preferences at a high degree of freedom, while limiting the range of each factor according to the target motor specifications.

The remainder of this paper is organized as follows: Section 2 describes the quantification process of individual driving styles through an analysis of real driving data. In Section 3, a personalization algorithm regulating the performance of EV acceleration under manual driving conditions is proposed based on the quantified driving behaviors derived from the analysis of personal driving data. Section 4 presents the simulation results to validate the proposed algorithm. Finally, Section 5 concludes this paper and states the plans for future work.

## 2. Quantification of Individual Accelerating Behavior

### 2.1. Driving Data Acquisition

**Figure 3:**
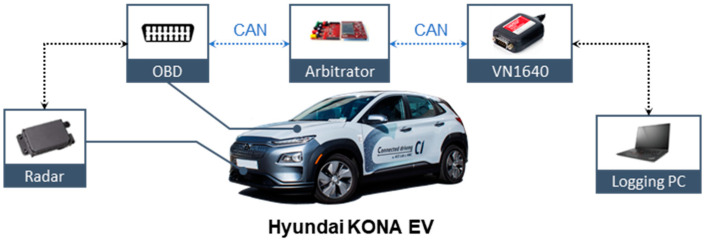
Vehicle configuration.

In order to design the personalization algorithm, drivers’ personal driving data were analyzed. The real driving data for driver modeling were collected through vehicle tests with various in-vehicle sensors. Figure 3 shows an overall structure of the in-vehicle data flow. The On-Board Diagnostics II (OBD II) was connected to the arbitrator using an Infineon TC237 through a CAN protocol. The arbitrator parsed the in-vehicle sensors’ data and transferred the data to the Vector tool. The measured data were logged using the Vector tool. The real-time acquisition was synchronized and recorded at 100 Hz.

A free-driving scenario refers to a situation in which the ego-vehicle runs freely without obstacles around it. In the free-driving scenario, since there is no disturbance affecting the speed of the ego-vehicle, the driver can operate the vehicle as they wish. Therefore, by analyzing the vehicle behavior in the free-driving scenario, it is possible to investigate the driver’s preferred driving style. However, even if there are no obstacles around the vehicle, driving behavior may be affected according to information pertaining to traffic or other external influences on the road. For example, the vehicle may start after being stopped by a stop signal from a traffic light, or it may accelerate again after decelerating during a right turn at an intersection. In such a situation, the driver’s propensity for acceleration, deceleration, and stopping can change.

In this paper, we focus on the situation when the vehicle accelerates from a stopped position under free-driving conditions. Individual driver characteristics during acceleration are then used to personalize the acceleration behavior of the EVs through quantification in Section 3. When the vehicle accelerates under these conditions, the situation in which the driver feels the acceleration most is when the vehicle starts from a standstill. In fact, when the vehicle starts from a standstill, the driver may momentarily feel uncomfortable due to the inertia of the vehicle and the traction force of the motor. In addition, there are differences in individual driver preferences for such a feeling of acceleration.

**Figure 4:**
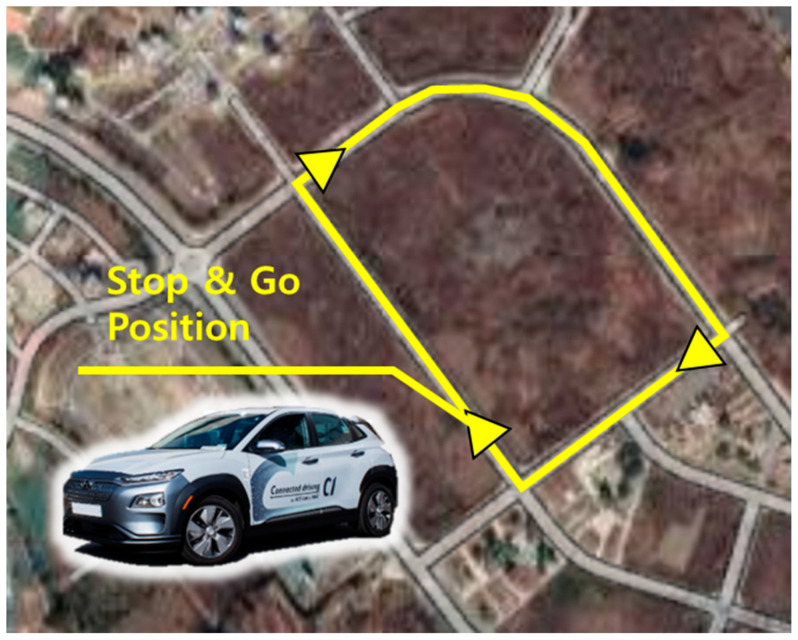
Driving scenario for the free-driving scenarios.

The driving route was set, as shown in Figure 4, and the driving data were acquired to investigate the characteristics of the starting from a stop states of each driver. The target route is located at a planned city in Incheon, South Korea, and the number of moving vehicles is very small, so the driving experiment constraints can be freely set. A total of five drivers performed data acquisition through the same scenario. Each driver repeatedly stopped and started while following the route. There were three intersections, and by continuing to turn right, the drivers could travel the same route indefinitely. The stopping point was each time a vehicle crosses an intersection on the target route. At that time, the driver faced a straight path and was able to accelerate freely. Furthermore, since there were no obstacles in front, the individual driving characteristics of the driver appear through the behavior of the vehicle. The reason why the data acquisition was not performed on a public road but proceeded under a restricted condition is that the driver’s behavior can change even if the driving scenario changes only slightly. Therefore, if the driving environment changed, the variability of the driver’s driving style increases, and it makes no sense to compare it from driver to driver.

### 2.2. Driving Data Analysis

#### 2.2.1. Initial Acceleration Section

**Figure 5:**
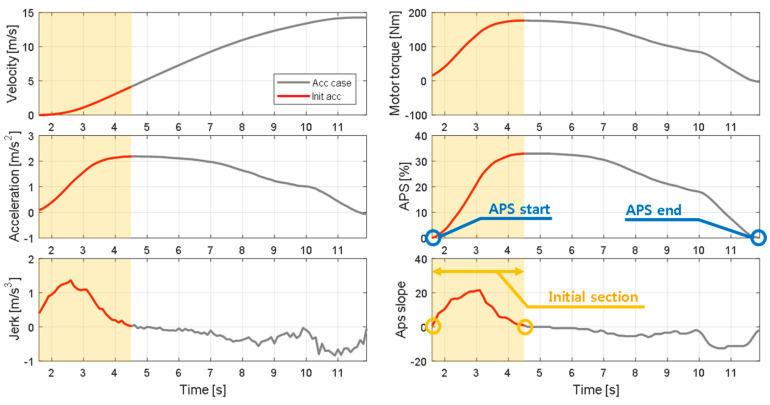
Definition of the initial acceleration section.

To effectively analyze the driving data acquired when the vehicle starts from a standstill in a free driving scenario, specific conditions are defined. The acceleration section and initial acceleration section are defined in all driving data. Figure 5 highlights the acceleration section for six physical vehicle states. The acceleration section is defined based on the driver’s APS operation. The acceleration section refers to the section from when the driver starts to operate the APS until it is finished. This definition is used to filter out acceleration situations that do not reflect the driver’s intention, such as vehicle inertia or acceleration due to driving downhill. Within this defined section, the motor generates propulsion torque through the driver’s APS operation, thereby causing the vehicle to accelerate.

The situation in which the feeling of acceleration felt by the driver in the acceleration section is dominant is during the initial acceleration. Therefore, we defined the initial acceleration section as the section in which the slope of the APS is positive. The initial acceleration section is considered as a transition period for obtaining the desired acceleration by the driver. In this section, the driving torque gradually increases until reaching a peak, and the vehicle acceleration also reaches a peak. During this process, the driver feels a great sense of acceleration and jerk that is not felt in other acceleration scenarios.

#### 2.2.2. Driving Features in the Initial Acceleration Section

**Figure 6:**
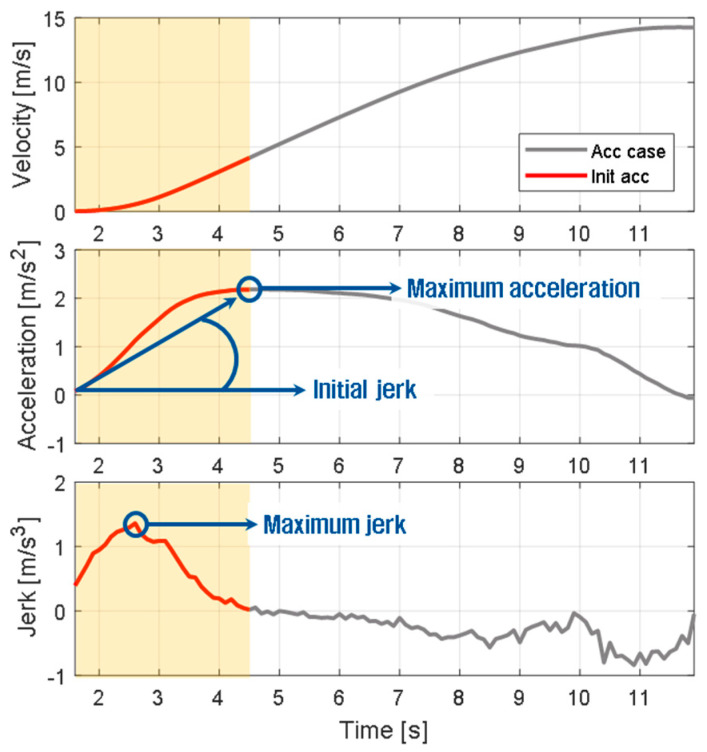
Driving feature distribution according to ego-vehicle velocity.

Based on data from the initial acceleration section, the driving features were defined. The driving features include physical values that can effectively express the initial acceleration tendencies of the vehicle. Three driving features were defined: maximum acceleration, maximum jerk, and initial jerk, as shown in Table 1. The maximum acceleration determines the driving feel in a situation where the speed is continuously increasing, and the value related to jerk determines the driving feeling during the initial period. The maximum acceleration is the peak value of acceleration that is maintained until the desired speed is reached. The maximum jerk is the maximum slope of acceleration. The initial jerk refers to the average jerk in the initial acceleration section. Figure 6 presents graphs of the driving features.

### 2.3. Quantification of the Individual Driving Features

**Figure 7:**
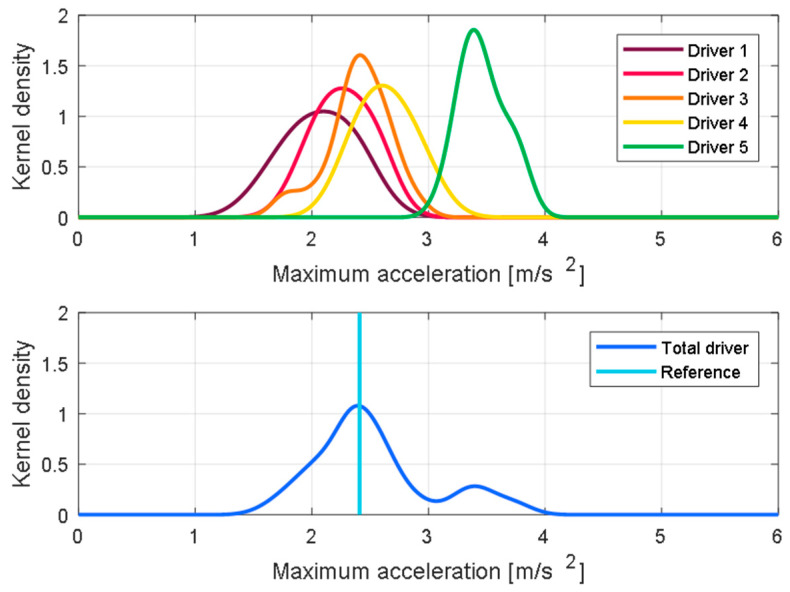
KDE of the maximum acceleration for each driver and the total number of drivers.

The determined driving features were statistically analyzed for each driver. To effectively compare the actual driving data for each driver, a non-parametric density estimation method, Kernel Density Estimation (KDE), was used. Due to the nature of the driving data, based on the driver’s simple change of mind, there is a lot of uncertainty that needs to be expressed parametrically. One driver characteristic, the kernel density function of the maximum acceleration, appears differently for the five drivers, as seen in the first graph in Figure 7. The fact that the distribution for each driver is in different locations means that the preferred values for each driver are different.

To express the distribution of the driving feature as one quantitative value, we used the median of the data distribution. The median value of the data has the advantage of minimizing the influence of the extreme values, compared to the commonly used arithmetic mean. In addition, to determine the standard of distribution of each driving feature, we checked the feature distribution of all the drivers and defined its median value as the reference value, as shown in the second graph of Figure 7. The median values of maximum acceleration for each driver and the reference value of the maximum acceleration are listed in Table 2.

**Figure 8:**
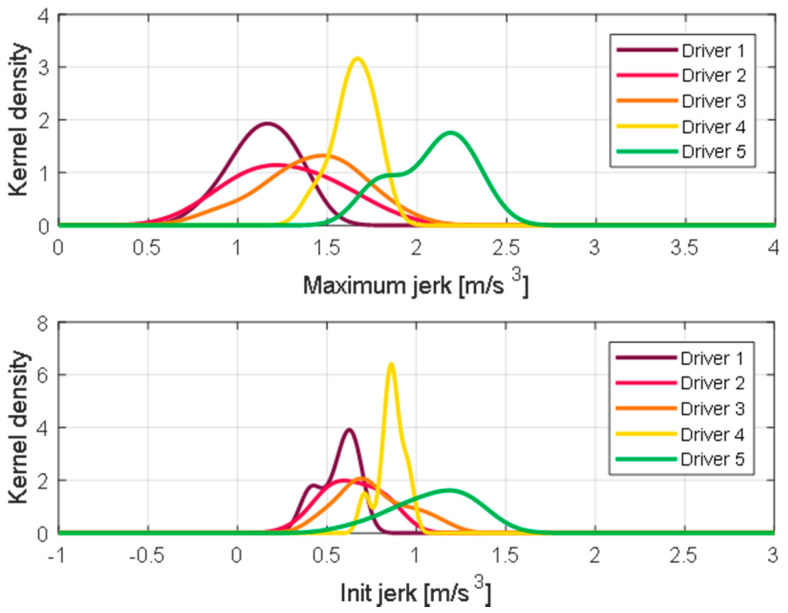
KDE of maximum jerk and initial jerk during the initial acceleration section.

For each driver, a relative driving tendency may be estimated based on the reference value. For example, the 3rd driver has a characteristic value most similar to all drivers, whereas the 5th driver prefers an exceptionally high maximum acceleration. Figure 8 presents the KDE distribution for each driving feature and each driver. For all driving features, drivers show similar trends and differences. The 5th driver shows the largest value for all driving features, and the 1st driver shows the smallest value.

**Figure 9:**
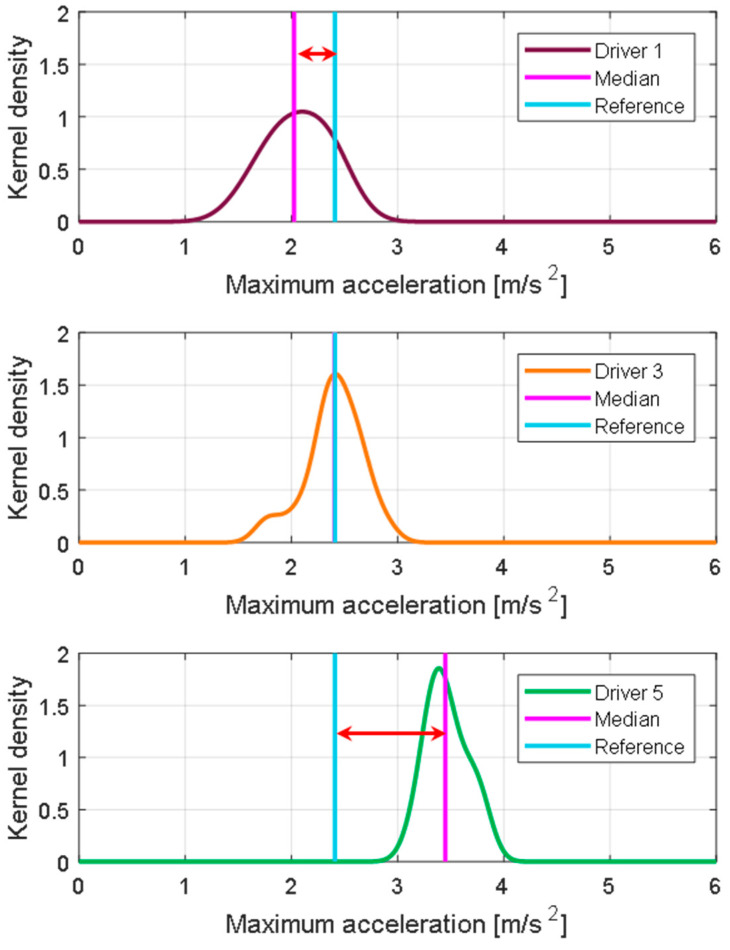
Feature deviation of maximum acceleration.

To effectively express the differences in propensity between drivers, the features were relatively compared. Figure 9 shows differences in the maximum acceleration for three drivers. The 3rd driver has the same tendency as the reference and can be considered a reference driver, whereas the 1st and 5th drivers show a distinct difference. If this difference in propensity is expressed as an absolute value, it is difficult to analyze it accurately, and as such it is not suitable for use in future applications.

The driving feature deviation is defined as each driver’s median value over the total driver’s reference value, based on the following equation:

(1) $$\sigma_{MaxAcc}\left(i_{driver}\right)=\frac{Med_{MaxAcc}\left(i_{driver}\right)}{Ref_{MaxAcc}}$$

All driving feature deviations calculated using this equation are shown in Table 3. When evaluating the driving tendency of each driver based on the maximum acceleration, it can be said that the 1st driver has a tendency 0.84 times that of the total driver value.

## 3. Personalization of EV Accelerating Performance

### 3.1. Acceleration Behavior of EVs

#### 3.1.1. Accelerator Pedal Operation

The longitudinal behavior of EVs is a result of traction control and braking control, according to human-drivers’ pedal operations [58]. In particular, acceleration behavior is dependent on traction control, which is related to the accelerator pedal. The accelerator pedal is controlled according to the driver’s intention according to the situation, and as a result, the longitudinal behavior of the vehicle appears. Therefore, when the acceleration tendency of the vehicle was analyzed in Section 2, the situation was defined according to the accelerator pedal.

In this study, the accelerator pedal is assumed to be the control input that the driver manipulates to achieve the desired longitudinal behavior of the vehicle. In other words, the driving style appears only through the behavior of the vehicle, and the accelerator pedal is only a variable that varies depending on the style.

#### 3.1.2. Target Motor Torque Calculation

**Figure 10:**
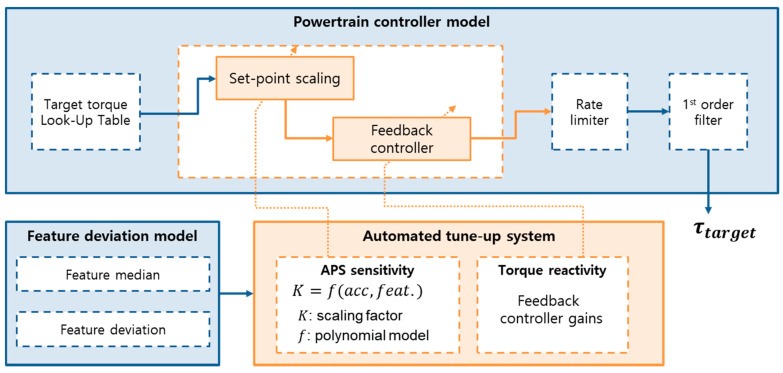
Target torque adjustment according to the feature deviation model.

The accelerator pedal has a decisive influence on setting the target torque within the motor controller. The simplified calculation process of the target motor torque is shown in Figure 10. The initial target torque is determined by LUTs using the vehicle state and Accelerator Pedal Sensor (APS) as inputs. Since the APS is directly adjusted by the driver, it can be said that the initial target torque reflects the driver’s intention. The initial target torque is used for motor control after a correction is made using a rate limiter and filter to ensure the driving stability of the EV. In this process, if the driver’s tendencies can be reflected in more detail, it would be possible to improve the driving comfort of an individual driver.

### 3.2. Personalization of the Target Motor Torque

We designed the personalization algorithm of the target motor torque to be used between the LUT of the initial target torque and the rate limiter. The algorithm consists of set-point scaling and the feedback controller. The set-point scaling factor primarily corrects the magnitude of the target torque according to the driver. The feedback controller then manipulates the transient behavior of the target torque by adjusting the reactivity of the controller.

#### 3.2.1. Set-Point Scaling Factor

The set-point scaling factor is calculated based on the vehicle’s acceleration and the feature deviation of each driving feature, as in the following equation.

(2) $$K=f\left(a,\;\sigma_{feature}\left(i_{driver}\right)\;\right)$$

**Figure 11:**
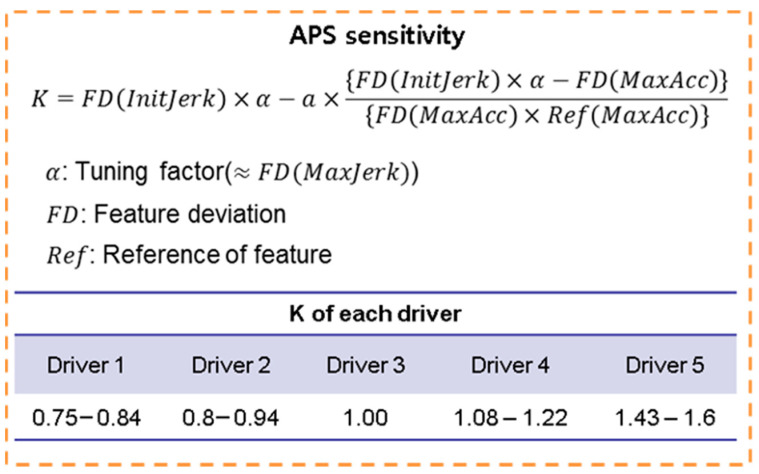
Configuration of the scaling factor model.

The scaling factor takes the role of modulating the initial target torque according to the driver’s preference. The maximum acceleration is the dominant limiting factor used to determine the value of the scaling factor, as they are both related to APS sensitivity. The APS sensitivity refers to how the absolute value of the APS affects the target torque. As shown in Figure 11, the scaling factor is affected by the vehicle’s acceleration and the predefined driving features. The range of the scaling factor values for each driver is summarized in the figure. The deviations for each driving feature are predefined values, so the current acceleration affects the change in value within a set range. Similar to the results of the driver data analysis, the scaling factor for the 5th driver is the largest because the 5th driver prefers more aggressive acceleration.

#### 3.2.2. Feedback Controller

The feedback controller ultimately affects the jerk by adjusting the dynamics of the required torque. The feedback controller was designed as a PI controller, and each P gain and I gain are scheduled according to the driver. In the case of a driver who wants more aggressive acceleration, the two gains become larger, and for a slightly milder driver, the gains decrease. Specifically, the scaling factor of the 1st driver is reduced compared to the general driver, and as the dynamics are slowed by the feedback controller the vehicle’s response to the acceleration input is slowed.

## 4. Validation

### 4.1. Simulation Environment

To validate the personalized motor controller, a simulation environment including an ego-vehicle was modeled based on the target vehicle, a Hyundai KONA EV, using the vehicle simulation software IPG CarMaker. IPG CarMaker enables detailed model configurations at the vehicle powertrain level, as shown in Table 4. A model was built in this simulation environment using all the specifications of the target vehicle, and a torque map was implemented.

### 4.2. Simulation Process

**Figure 12:**
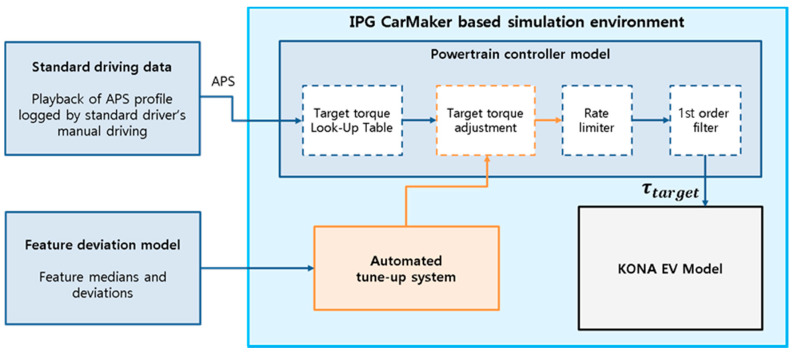
Simulation environment for the automated tune-up system.

The IPG CarMaker-based simulation environment was integrated with the actual driving data to validate the personalized target torque of the traction motor controller. The purpose of this simulation was to prove that when the same APS profile was used as an input, the behavior of the vehicle changed according to the driver due to personalization of the target torque, as shown in Figure 12.

In the case of a general EV with no personalization algorithm, the driver controls the APS feedback to achieve the desired longitudinal behavior. As a result of the APS feedback, the aggressive drivers need more dynamic pedal operation than the milder drivers. However, if the performance of the EV motor controller is automatically set to suit a specific driver, even an aggressive driver should be able to obtain their desired vehicle behavior using only normal pedal operation. To verify this assumption, the most average driver’s APS profile was used as the input for these simulations. Here, we use the 3rd driver’s profile as the average driver, based on the results of the driving data analysis.

For an identical APS profile, the personalization algorithm corrects the torque using the scaling factor and feedback controller set for each driver. The result is then compared to the actual driving data of the corresponding driver to evaluate the torque correction performance. For example, the personalization algorithm configured to the mildest 1st driver and the APS profile of the normal 3rd driver are integrated in order to replicate the driving behavior of the 1st driver’s actual data.

### 4.3. Simulation Results

#### 4.3.1. Results of Motor Torque Personalization

The proposed algorithm was confirmed to reproduce different vehicle behaviors through the scaling factor and the feedback controller, as shown in Figure 12. This figure presents the simulation results for the five driver settings for the algorithm in the acceleration section, as defined in Section 2. As explained in the simulation process, the APS profile is identical for the five simulation results. However, due to the influence of the personalization algorithm, the motor torque results display different behaviors. In the case of the 5th driver, who preferred a large acceleration in real life, the algorithm produces a significantly higher acceleration and jerk compared to the results of the other drivers.

**Figure 13:**
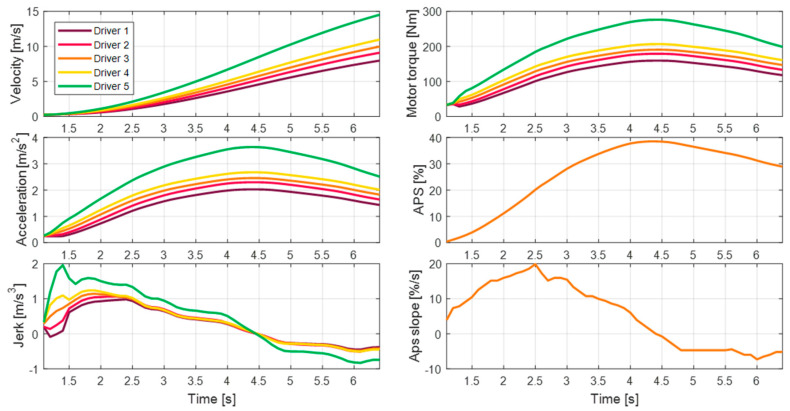
Acceleration behavior by target torque personalization for identical APS profiles.

The difference in the results of each driver is similar to the driving feature deviation values determined through the quantification of driving behavior. In the case of maximum acceleration, the feature deviation of the 5th driver is 1.43. The feature deviation of the 5th driver derived from the case shown in Figure 13 is 1.51, and the error is small. As a result, through target torque personalization, it was confirmed that various initial acceleration behaviors could be replicated from an identical APS profile.

#### 4.3.2. Comparison with Experimental Data

**Figure 14:**
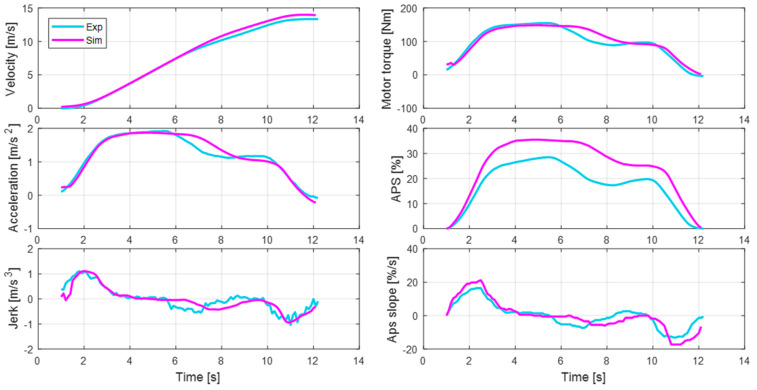
Comparison of the acceleration behavior between the experiments and simulations of the 1st driver.

Figure 14 compares the experimental data acquired by human driving and the simulation results to validate the proposed algorithm. The experimental data refers to the actual driving results of the 1st driver, driving a target vehicle that is not personalized. On the other hand, the simulation data refers to the results of the motor control that was personalized for the 1st driver using the 3rd driver’s APS profile. Therefore, each APS profile shows different behaviors.

Since there are two APS profiles in which different drivers drove under similar driving conditions, not only the magnitude but also the overall behavior are different. For example, there is a difference in both the period maintaining the maximum jerk in the initial acceleration section, and in the period maintaining the maximum acceleration. Despite the different APS profiles, the acceleration in the simulation results that was corrected through the personalization algorithm is similar to that of the experimental results. The acceleration behavior in the initial acceleration section is particularly well replicated, though the speed of the vehicle and the torque of the drive motor also appear very similar.

**Figure 15:**
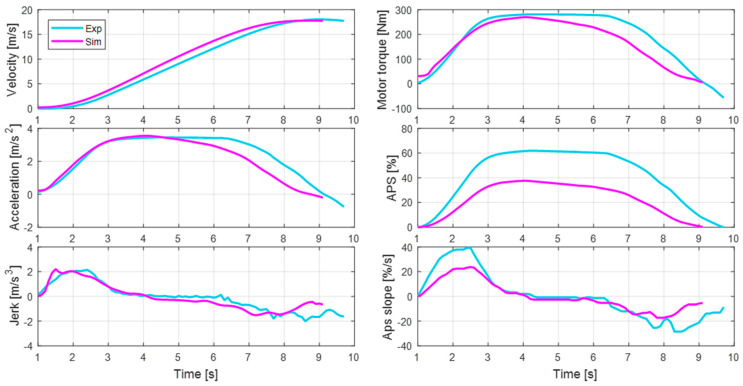
Comparison of acceleration behavior between the experiments and simulations of the 5th driver.

Figure 15 represents the validation results for the 5th driver’s case. As with the previous analysis, the effectiveness of the personalization algorithm is well revealed. One difference from the previous results is that the APS profile of the experiment appears more intense due to a difference in the driver’s disposition. Although the behavior of the APS displays a larger difference than for the 1st driver, the acceleration of the initial acceleration section is still well replicated. The difference in behavior after the initial acceleration period is due to a change in the APS and is not related to the proposed algorithm.

#### 4.3.3. Statistical Evaluation

**Figure 16:**
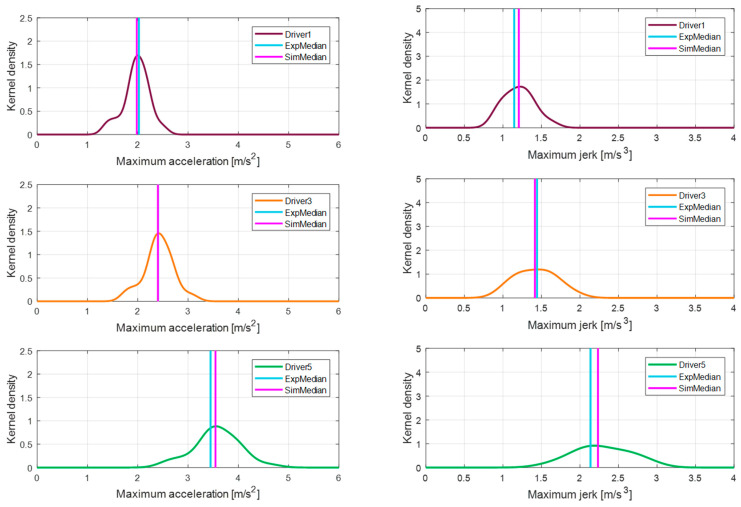
Comparison of the MaxAcc and MaxJerk distributions between the experiments and simulations.

To quantitatively evaluate the acceleration behavior in the simulations regarding the performance of the proposed algorithm, a statistical analysis was performed in the same manner as for the driving behavior quantification process. The personalization algorithm for each driver was simulated for all acceleration scenarios under which the actual drivers were driving, and the KDE was applied to the results. Figure 16 represents the KDE distribution and the median of the driving results of the maximum acceleration and maximum jerk for three drivers.

Table 5 summarizes the personalization accuracy results of the maximum acceleration and maximum jerk for all drivers. The personalization accuracy was evaluated based on the absolute error and percentage error of the medians. First, in the case of the 3rd driver, since they are the standard driver, the results were not affected by the APS or personalization algorithms, so the median of the maximum acceleration was the same in both the experimental and simulation results. Note that though a very small error of 0.17% occurred, it was due to modeling errors.

The maximum acceleration of the 1st driver was correctly replicated by the proposed algorithm, though there was a small error of 2.18% between the two medians. Finally, in the result of the 5th driver, the driver’s preference was exceptionally different, so the replication performance was slightly lower than the other two drivers, but still displayed a high accuracy (error of 2.81%).

## 5. Conclusions

This paper quantitatively characterized individual drivers’ features and implemented a personalization algorithm of the EV’s accelerating performance under a driver’s manual driving. The driver characteristics quantification algorithm derives the expected statistical value for each driving feature. This approach focuses on determining one representative value for one driving feature representing a driver’s personal preference. The driving features are physical values that can express longitudinal driving behaviors among vehicle states, and display different tendencies depending on the driving pattern. In order to personalize longitudinal vehicle behavior, the quantified driving features are applied to the traction motor controller of the EVs. The traction motor controller calculates the target torque based on the accelerator pedal position in order to reflect the driver’s intentions. In this process, we utilized the quantified driving features to calculate the correction factors of the target torque. This driver-specific correction provided satisfactory propulsion performance for each driver. The proposed algorithm was then validated through simulation studies consisting of a precise EV model. The validation results confirmed that the proposed motor controller could reproduce different acceleration behaviors for identical accelerator pedal inputs. Through a comparison between the experimental and simulation data, the distributions of each driving feature displayed a high similarity. In the case of the maximum acceleration, errors in the distribution medians were lower than 3%.

The proposed target torque personalization satisfactory improves individual driving. The target torque table is generally calibrated through experiments to satisfy standard acceleration performance. The previous research introduced look-up tables of the correction factors to adjust the torque within a range of motor specifications. The limitations of the previous research were that the correction look-up tables depended on the motor’s operation range and showed discrete correction results. To overcome these limitations, the proposed algorithm automatically calculates the correction factors through personal driving data analysis. Furthermore, to prevent stability problems, the proposed correction factor is limited according to the target motor specifications. The adjustment of the EV’s output torque greatly affects not only vehicle performance but also driving safety. For example, if the driving tendency a driver selected as a personalization target is excessively aggressive, it is not necessary to compensate for the driver unconditionally, but to offer support within the range that guarantees driving safety. Even if it is possible to tune the motor to achieve higher acceleration according to the specifications of the EV, safety must be ensured with a certain margin, and a service that recommends improving driving style rather than personalization to drivers outside the range must be provided. In addition to the acceleration performance discussed in this paper, personalization in the ADAS field has safety as the top priority, and research is being conducted to provide a personalized function for each driver within the possible range.

## Figures and Tables

**Table 1:** Specification of the vehicle (Hyundai KONA).

Driving Feature	Description
Maximum acceleration	Maximum acceleration maintained until the desired speed is reached
Maximum jerk	Maximum jerk during initial acceleration section
Initial jerk	Average jerk during initial acceleration section

**Table 2:** Median values of the driving features.

Driver #	Max. Acc (m/s^2^)	Max. Jerk (m/s^3^)	Initial Jerk (m/s^3^)
Driver 1	2.02	1.15	0.59
Driver 2	2.26	1.27	0.64
Driver 3	2.41	1.44	0.72
Driver 4	2.60	1.64	0.86
Driver 5	3.45	2.14	1.13
Total Drivers	2.41	1.44	0.71

**Table 3:** Feature deviation values of the driving features.

Driver #	Max. Acc (−)	Max. Jerk (−)	Initial Jerk (−)
Driver 1	0.84	0.79	0.83
Driver 2	0.94	0.88	0.90
Driver 3	1.00	1.00	1.01
Driver 4	1.08	1.14	1.21
Driver 5	1.43	1.48	1.60

**Table 4:** Specification of the vehicle (Hyundai KONA).

Vehicle Parameter	Value	Powertrain Parameter	Value
Unloaded weight	1685 kg	Maximum torque (Motor)	395 Nm
Length	4180 mm	Maximum power (Motor)	150 kW
Width	1800 mm	Maximum rpm (Motor)	11,000 rpm
Height	1570 mm	Inertia (Motor)	0.028 kg·m^2^
Driving axle	Front driven	Capacity (Battery)	180 Ah 64 kWh
Tire specification	215/55 R	Idle voltage (Battery)	353 V
Tire radius	17 inches	Maximum power (Battery)	150 kW

**Table 5:** Evaluation of the feature similarities between the experiments and simulations.

	Maximum Acceleration		Maximum Jerk	
Driver #	Error (m/s^2^)	Error (%)	Error (m/s^3^)	Error (%)
Driver 1	0.0440	2.1728	0.0600	5.2310
Driver 2	0.0180	0.7940	0.0660	5.1969
Driver 3	0.0040	0.1663	0.0270	1.8711
Driver 4	0.0150	0.5769	0.0890	5.4268
Driver 5	0.0970	2.8124	0.0970	4.5391
